# Blocking central pathways in the primate motor system using high-frequency sinusoidal current

**DOI:** 10.1152/jn.00347.2014

**Published:** 2014-12-04

**Authors:** Karen M. Fisher, Ngalla E. Jillani, George O. Oluoch, Stuart N. Baker

**Affiliations:** ^1^Institute of Neuroscience, Medical School, University of Newcastle upon Tyne, Newcastle upon Tyne, United Kingdom; and; ^2^Institute of Primate Research, National Museums of Kenya, Karen, Nairobi, Kenya

**Keywords:** corticospinal, high-frequency block

## Abstract

Electrical stimulation with high-frequency (2–10 kHz) sinusoidal currents has previously been shown to produce a transient and complete nerve block in the peripheral nervous system. Modeling and in vitro studies suggest that this is due to a prolonged local depolarization across a broad section of membrane underlying the blocking electrode. Previous work has used cuff electrodes wrapped around the peripheral nerve to deliver the blocking stimulus. We extended this technique to central motor pathways, using a single metal microelectrode to deliver focal sinusoidal currents to the corticospinal tract at the cervical spinal cord in anesthetized adult baboons. The extent of conduction block was assessed by stimulating a second electrode caudal to the blocking site and recording the antidromic field potential over contralateral primary motor cortex. The maximal block achieved was 99.6%, similar to findings of previous work in peripheral fibers, and the optimal frequency for blocking was 2 kHz. Block had a rapid onset, being complete as soon as the transient activation associated with the start of the sinusoidal current was over. High-frequency block was also successfully applied to the pyramidal tract at the medulla, ascending sensory pathways in the dorsal columns, and the descending systems of the medial longitudinal fasciculus. High-frequency sinusoidal stimulation produces transient, reversible lesions in specific target locations and therefore could be a useful alternative to permanent tissue transection in some experimental paradigms. It also could help to control or prevent some of the hyperactivity associated with chronic neurological disorders.

many experimental and clinical applications require the control of neural activity. Electrical stimulation is capable of increasing the overall level of activity as well as eliciting action potentials in the stimulated elements at times precisely defined in the submillisecond range. However, in some cases it also can be important to reduce or abolish activity in a chosen pathway. Clinically, this could ameliorate symptoms caused by pathological over activity (e.g., spasticity); experimentally, it allows measurement of responses in which contributions from the blocked pathway have been excluded.

The simplest approach to block neural conduction is to disrupt the neural center or axon bundle by making a lesion. This may involve direct surgical transection, thermocoagulation, focal ischemia, or irradiation. The irreversible nature of these interventions may be an advantage in some clinical situations, where a permanent solution to overactivity is desired. However, in other cases it would be preferable to induce only transient blocking of activity. For example, in neuroprosthetic approaches to restoring bladder function following spinal cord injury, the pudendal nerve must be blocked to induce sphincter relaxation at the same time as bladder contraction, producing coordinated micturition. At all other times, maintenance of normal tone in the pudendal nerve is beneficial, because it maintains continence. In experimental studies, recordings made before and after a pathway is lesioned are typically compared (Alstermark et al. 1999); however, the permanent nature of the lesion means that only one set of data can be gathered from a single experimental animal.

Currently available methods for reversible neural inactivation include focal cooling and pharmacological blockade. Cooling requires introduction of a relatively bulky probe, which may produce permanent damage; it also can be difficult to control the extent of cooled tissue. Moreover, the onset and offset time course is typically on the order of minutes, and long-term use of cooling can cause local damage (Lomber 1999). Pharmacological methods such as channel blockers (local anesthetics; e.g., lignocaine) and inhibitory receptor agonists (e.g., muscimol) have limited spatial specificity due to diffusion of the drug within the tissue. Although they may have an onset over minutes, the effects usually wear off over several hours.

Electrical stimulation has long been known to block conduction in neural tissue when high frequencies are used (Bowman and McNeal 1986; Rosenblueth and Reboul 1939; Tanner 1962; Wedensky 1903). This principle has been tested extensively in peripheral nerves with the use of high-frequency (HF) sinusoidal currents. The conduction block produced has a very rapid onset (tens of milliseconds) and is reversible with a similarly quick recovery time. These collective features may make it particularly suitable as a clinical intervention.

Peripheral nerve conduction block using HF sinusoidal stimulation was initially demonstrated in isolated muscle preparations in frogs (Kilgore and Bhadra 2004; Tanner 1962); complete block of all myelinated fibers could be achieved with stimuli at 3–5 kHz (Kilgore and Bhadra 2004), and block was reversed when the stimulation stopped. More recently, Joseph and Butera (2011) showed that block was not restricted to myelinated axons but also could encompass unmyelinated C-fibers. This required different stimulus parameters, raising the possibility of blocking selective subpopulations of fibers within a mixed nerve. Modeling studies have further suggested that large-diameter axons should have a lower threshold for blocking (Bhadra et al. 2007; Tai et al. 2005).

This technique has been applied to pudendal nerve for the treatment of detrusor sphincter dyssynergia (Bhadra et al. 2006; Tai et al. 2004) and to vagus nerve for treatment of obesity (Camilleri et al. 2008). Optimal frequencies for cat pudendal nerve were in the 6- to 10-kHz range. Bhadra and Kilgore (2005) investigated blocking of sciatic nerve in rats; the optimal frequency was 10 kHz, the lowest tested. In macaque monkey median nerve, the most effective frequency was 20–40 kHz (Ackermann et al. 2011b); frequencies around 10 kHz produced tetanic activation, not blocking. Across different subjects, Ackermann et al. (2011) described a positive correlation between nerve diameter and block threshold. The higher optimal frequencies found in their study may therefore be related to the larger nerve diameter in monkey (3–4.1 mm) compared with the smaller nerves in other species.

There is some debate over the mechanism of HF blocking; however, the effect is known to be restricted to the area close to the electrodes delivering the HF sinusoid, since stimulation of a position more distal on the nerve results in normal muscle activation (Kilgore and Bhadra 2004). Modeling studies suggest that the stimulus produces a steady-state depolarization of a broad section of membrane directly underneath the blocking electrode (Bhadra et al. 2007; Kilgore and Bhadra 2004; Williamson and Andrews 2005). Tai et al. (2005) proposed that the most important effect is the relatively high activation of potassium channels consequent on the depolarized membrane potential.

Thus far, most attention has been focused on HF blocking of peripheral nerves, but similar approaches could find widespread utility in central pathways. There is evidence that deep brain stimulation of the subthalamic nucleus for treatment of Parkinson's disease acts mainly by blocking endogenous activity, and not by augmenting activity via stimulation (Bellinger et al. 2008; Jensen and Durand 2009). However, the stimulus frequencies (∼130 Hz) and waveforms (biphasic square pulses, 60- to 150-μs duration per phase) are very different from the sinusoidal stimuli in the kilohertz range used by work on peripheral nerve. Elbasiouny and Mushahwar (2007) used computer modeling to describe the effect on motoneuron firing of a HF sinusoidal current delivered through a nearby microwire electrode. Blocking was achieved with frequencies comparable to those used in peripheral nerve (5 kHz); the mechanism appeared to be sustained depolarization of the initial axon segment.

Spinal cord stimulation via a chronically implanted device is routinely used for the treatment of chronic pain. Such devices normally stimulate at low frequencies (between 30 and 100 Hz), but recently there has been interest in using kilohertz range stimuli. A large clinical trial has demonstrated promising effects by delivering 10-kHz stimuli to electrodes with tips in the epidural space of the thoracic segment (Al-Kaisy et al. 2014). The authors report a significant and sustained reduction in pain scores over a 24-mo period in the majority of patients. In contrast, however, results from basic science studies carried out in rats are conflicting (Shechter et al. 2013; Song et al. 2014).

To date, few studies have investigated the potential of HF sinusoidal stimuli to block conduction in central axon tracts. Aside from possible biophysical differences between central and peripheral axons, an important methodological difference is the type of electrode used to deliver the stimulus. All studies of peripheral nerve use cuff electrodes, which ensheath the nerve, focusing the current along the axon fascicles. By contrast, stimuli to central axon pathways are typically delivered via metal electrodes, insulated except for the tip. This allows stimuli to be delivered to deep target tracts, located via stereotaxic coordinates and electrophysiological response signatures, without damage to overlying neural structures. Activity block has been achieved with the use of tungsten microwire electrodes placed directly within the rat sciatic nerve (Ackermann et al. 2010b), but it remains unclear whether HF currents delivered through fine-tipped electrodes will be capable of producing sufficient blocking of central pathways to be useful, either clinically or experimentally.

In this study, we characterized the effect of HF sinusoidal stimuli on the primate corticospinal tract. Stimuli were delivered through a single sharp metal electrode and a distant reference. We found that near-complete blocking of fast corticospinal conduction could be obtained, with a rapid onset; the effect was reversible, also over a rapid timescale. We further demonstrate these findings in other primate central pathways and suggest that this method could have widespread uses, both to generate transient reversible lesions in animal studies and potentially to treat neurological disease caused by excess activity in a defined central tract.

## METHODS

### 

#### Anesthesia and surgical preparation.

The main experimental series was performed in five anesthetized healthy adult male baboons (*Papio anubis*; 22.5–26 kg) as part of longer studies unrelated to the present report. All animal procedures were approved by the local ethics committee of the Institute of Primate Research, Nairobi, Kenya. Animals were initially anesthetized with intramuscular injection of ketamine (10–12 mg/kg) and xylazine (0.5–0.75 mg/kg). After intubation and insertion of an intravenous line, deep general anesthesia was maintained with inhaled halothane (1–2% in 100% O_2_) and continuous intravenous infusion of fentanyl (1–4 μg·kg^−1^·h^−1^). The animals were artificially ventilated using a positive pressure ventilator. Initial surgical preparation included a tracheotomy (which replaced the originally inserted endotracheal tube, providing more stable long-term airway protection) and insertion of a central arterial line for continuous blood pressure measurement via the carotid artery on one side. Methylprednisolone (initial loading dose of 30 mg/kg, followed by infusion of 1–7 mg·kg^−1^·h^−1^) was administered to reduce cerebral edema, and Hartman's solution (1.5–6.5 ml·kg^−1^·h^−1^) was administered to ensure fluid balance. The urethra was catheterized to prevent urinary retention, and temperature was maintained using a heating blanket supplied with thermostatically controlled warm air. Anesthetic monitoring included heart rate, arterial blood pressure, pulse oximetry, end-tidal CO_2_, and core and peripheral temperatures.

The head was fixed in a stereotaxic frame, and a craniotomy over the left motor cortex (M1) was made to give access for epidural field potential recording. A laminectomy was performed to expose spinal segments T1–C5, and the spinal dura was removed to allow access to the cord. The vertebral column was clamped at the high thoracic level. In two animals a second craniotomy was opened on the right side and a small piece of M1 removed for an in vitro experiment unrelated to the present report.

Anesthesia was then switched to a combination of midazolam (0.4–2.4 mg·kg^−1^·h^−1^), ketamine (0.1–0.8 mg·kg^−1^·h^−1^), and fentanyl (2.9–11.2 μg·kg^−1^·h^−1^) for the electrophysiological recordings, because we have previously found that this yields stable anesthesia but leaves central nervous system circuits more excitable. Slow rising trends in heart rate or blood pressure, or more rapid rises in response to noxious stimuli, were taken as evidence of lightening anesthesia; supplemental doses were then given and infusion rates adjusted accordingly. During spinal stimulation protocols, neuromuscular blockade was initiated by giving an intravenous bolus of atracurium (10–15 mg per injection); this was repeated approximately every hour as required to maintain block.

At the end of each experiment, the animals were killed by overdose of anesthetic; where tissue was to be harvested for histological analysis, the animal was perfused through the heart with phosphate-buffered saline followed by fixative.

Further experiments were carried out in one adult male macaque monkey (17.7 kg), with ethical approval from the Newcastle University Animal Welfare and Ethical Review Body and under appropriate licenses from the UK Home Office. This animal had previously been used for a chronic series of experiments on visual pathways, but his motor system remained undisturbed. The animal was initially anesthetized with an intramuscular injection of ketamine (10 mg/kg), and surgical procedures were carried out under sevoflurane (2–3.5%) with an additional infusion of alfentanil (12–15 μg·kg^−1^·h^−1^). A laminectomy was performed and the spinal dura removed to facilitate access to the T1–C5 segments. In addition, we made bilateral craniotomies over sensorimotor cortex for the purposes of recording cortical potentials. Physiological measures were monitored throughout the procedure as described in the experiments above. During recording, the anesthetic regimen was changed to an infusion of midazolam (0.9 mg·kg^−1^·h^−1^), ketamine (0.6 mg·kg^−1^·h^−1^), and alfentanil (12 μg·kg^−1^·h^−1^), and neuromuscular blockade was achieved with atracurium (0.7 mg·kg^−1^·h^−1^). At the end of this experiment, the animal was killed by overdose of anesthetic.

#### Electrophysiological recordings.

In four animals, we investigated the properties of HF blocking stimuli delivered through electrodes positioned in the spinal cord.

“Hatpin” electrodes were made by joining a sharpened stainless steel electrode (MicroProbes, Gaithersburg, MD; order code MS501G; shaft diameter 256 μm, tip diameter 3–4 μm, estimated exposed surface area 310 μm^2^, parylene-C insulated, tip impedance ∼10 kΩ) to Teflon-coated seven-strand stainless steel wire. This was insulated with epoxy adhesive, leaving an ∼3-mm insulated length of electrode protruding from the flat surface of the epoxy. One such electrode was inserted manually into a caudal section of the exposed spinal cord (approximate segmental level C6) on the right side, targeting the dorsolateral funiculus. Small adjustments were made using forceps to maximize the antidromic response observed over motor cortex following stimulation through this electrode, verifying its location within the dorsolateral funiculus, and it was then fixed in place using tissue glue. A standard stainless steel microelectrode (Microprobe SS30030.1A10; tip impedance <0.1 MΩ, tip diameter 2–3 μm, estimated exposed surface area 180 μm^2^) was then inserted into a more rostral region of the spinal cord (around C5 segment) with the use of a three-axis stereotaxic manipulator, allowing us to examine how effects from this electrode depended on the tip location.

**Fig. 1.**
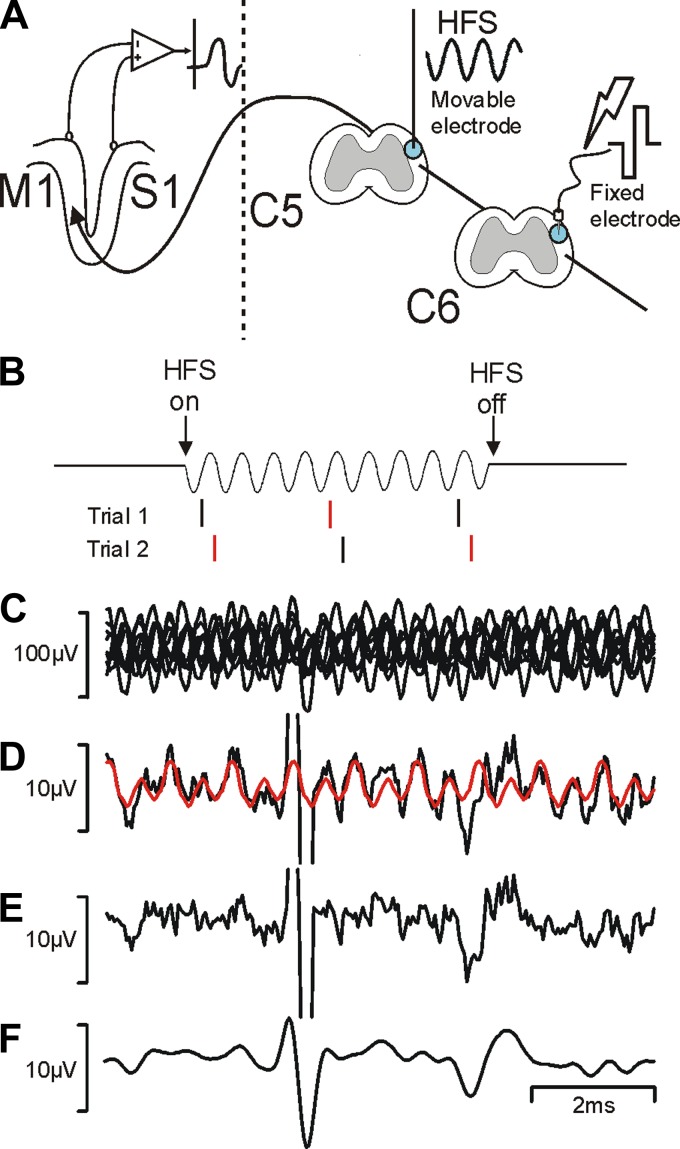
Experimental design. *A*: schematic showing the stimulation and recording sites used in the experimental protocol. HFS, high-frequency sinusoid; M1, motor cortex; S1, somatosensory cortex; C5 and C6, spinal segments. *B*: stimulus timing used to assist cancellation of stimulus artifact. Stimuli were delivered in phase (black) or out of phase (red) with the sinusoidal stimulus. *C*: overlain single sweeps (*n* = 8) of responses recorded from M1 following stimulation through the caudal electrode (500 μA) during sinusoidal stimulation through the rostral electrode (2 kHz, 1 mA). Note the large sinusoidal contamination, which is at opposite phases in successive sweeps. *D*: black line shows the average of traces in *C*; red line shows an estimate of the residual sinusoidal contamination produced by a cycle-triggered average from the prestimulus period. *E*: difference between black and red traces in *D*. *F*: trace shown in *E* after application of a digital low-pass filter (cutoff 1.7 kHz). Note the different scale bars in *C* and *D–F*.

A schematic diagram of the experimental setup is shown in Fig. 1*A*. Stimuli were delivered to the caudal fixed electrode using an isolated constant-current stimulator at 2 Hz (AM Systems, Carlsborg, WA; biphasic pulses 0.2 ms per phase, intensity 500 μA or 1 mA). The blocking stimulus was provided by a device that converted a voltage command signal to a constant-current isolated output (DS4 stimulator; Digitimer, Welwyn Garden City, UK). This device was calibrated using a 20-kΩ load resistor across the two output contacts; output voltage was then measured on an oscilloscope and confirmed to be as expected. Sinusoidal currents had a frequency between 2 and 10 kHz, intensity of 200 μA to 1 mA, and duration of 0.53 s. Throughout this report, intensity is given as the peak amplitude, i.e., the maximal positive or negative excursion relative to baseline, which equates to half the peak-to-peak amplitude. An epidural cortical recording was made over M1 with silver ball electrodes resting lightly on the dura (gain 5,000, bandpass 300 Hz-10 kHz). Command waveforms and stimulus delivery were controlled by Spike2 software and a Micro1401 interface (CED, Cambridge, UK), which also sampled waveform data to disk at 25 kHz, together with markers indicating stimulus occurrence times.

Antidromic corticospinal responses in M1 were elicited following stimulation through the caudal spinal electrode (fixed “hatpin”) at constant stimulus intensity. Stimulation through this electrode alone was interleaved with stimulation combined with HF sinusoidal stimulation through the rostral (movable) electrode. In most experiments, stimuli were given ∼10, 250, and 500 ms after the onset of the sinusoidal current. We averaged together responses to the various stimuli given at 250 and 500 ms after verifying that both yielded similar results. Recordings were carried out with the rostral electrode at multiple depths to determine the spatial properties of HF block in the spinal cord. At each recording site, an intensity and frequency series for the HF block was recorded. We also measured the extent of overlap between corticospinal fibers that could be activated from the two electrodes by measuring responses to stimulation of each alone (biphasic square current pulses, as described above) and both together (occlusion).

In one baboon, we tested the ability of HF stimulation to provide information on pathways contributing to a response of unknown origin. Stainless steel electrodes as described above were inserted into the right-side medial longitudinal fasciculus (MLF) and left-side pyramidal tract (PT) in the medulla, using the double-angle stereotaxic method described by Soteropoulos and Baker (2006). Final electrode placement optimized the responses in field potential recordings from the surface of M1 and the cervical spinal cord. Responses in the spinal cord were then recorded following trains of three stimuli to the PT; the effect on these responses of delivering sinusoidal blocking currents to the MLF was tested.

We further tested parameters of HF block in one macaque monkey. These experiments used metal microelectrodes (as previously described) that were implanted into the pyramidal tract (positioned as above) and dorsal column of the spinal cord (hatpin electrode, positioned to optimize somatosensory evoked potential in sensorimotor cortex). In addition to HF sinusoidal stimuli, we also tested HF square-pulse stimuli delivered to the PT; these were produced using a standard isolated constant-current stimulator (model 2100; AM Systems). This animal also had a bipolar nerve cuff implanted around the median nerve in the upper arm to allow direct stimulation of the nerve.

#### Measures to reduce sinusoidal stimulus artifact.

Delivering continuous sinusoidal stimulation posed a problem when making simultaneous recordings of electrical activity, as recorded signals were almost always contaminated by a large sinusoidal stimulus artifact. To reduce the impact of this, we designed the timing of test stimuli to be delivered at opposite phases of the sinusoidal current in successive trials (see Fig. 1*B*). Figure 1*C* shows an example of overlain single sweeps of recordings, which make clear the extent of the sinusoidal contamination and also illustrate the opposite phases of successive sweeps. Figure 1*D* shows an average of these waveforms; the artifact was much reduced by cancellation (note the scale bar is one-tenth of that in Fig. 1*C*). However, some residual contamination was still present.

Two signal processing methods were used to reduce the artefact further. First, we estimated a template for the sinusoidal contamination by constructing a cyclical average of the baseline (prestimulus) region. This averaged successive 1-ms-long sections of waveform; the cyclic average was then replicated over the entire average time course, both before and after the stimulus (red trace, Fig. 1*D*). We used a 1-ms cycle time for this process, because all sinusoidal frequencies tested were integer multiples of 1 kHz. The artifact template was then subtracted from the actual average (Fig. 1*E*). Part of the success of this method came from the fact that the same microprocessor-based system controlled the sinusoid generation and data capture, meaning that there was no drift between successive cycles of sinusoid and the data acquisition clock. Finally, we digitally low-pass filtered the response, using a cutoff frequency of 1.7 kHz, which was below the lowest stimulus frequency that we used (2 kHz). The filtered trace is shown in Fig. 1*F*.

**Fig. 2.**
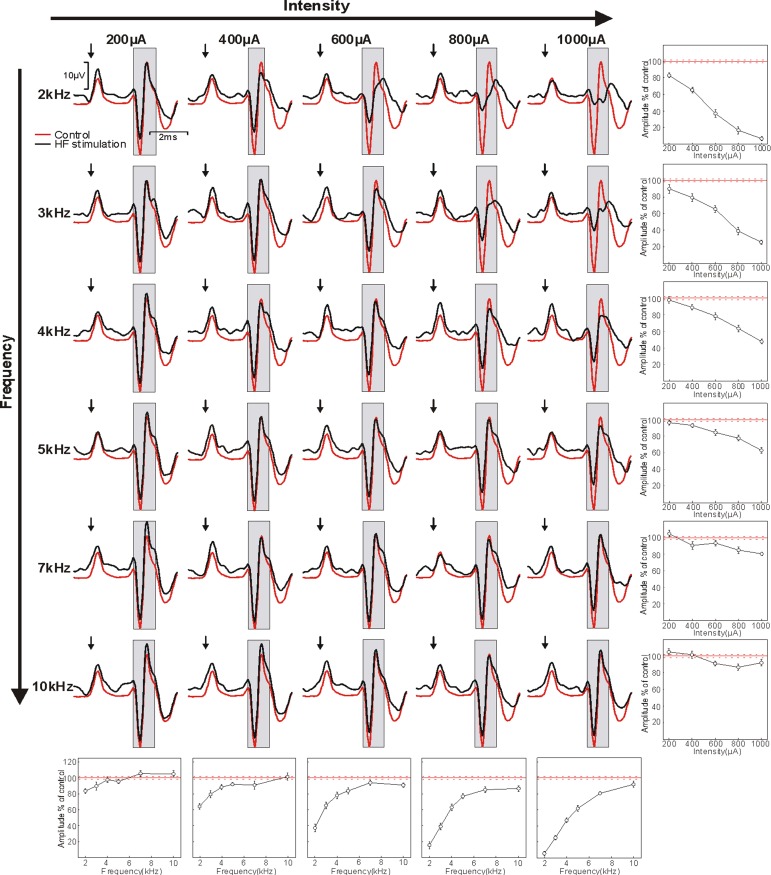
Blocking effects of HF sinusoidal stimulation on antidromic corticospinal potentials recorded over M1. HF sinusoidal stimuli were applied to the spinal cord rostral electrode with different combinations of frequency (rows; 2–10 kHz) and intensity (columns; 200–1,000 μA) at the same time as stimulation of the caudal electrode (500 μA, biphasic pulses). In each panel, the red line shows the response in the M1 epidural recording to the caudal electrode alone and the black line shows the response obtained during delivery of the sinusoidal blocking stimulus. Arrows mark the time of caudal stimulus delivery; gray boxes indicate the region over which the amplitude of the antidromic response was measured. Plots at the end of each row and column show how amplitude varied with intensity or frequency for fixed frequency or intensity, respectively. Amplitudes are expressed relative to the response to the caudal electrode alone (100%; solid red line). Error bars and dotted red lines indicate means ± SE. Data were recorded in *baboon M*.

**Fig. 3.**
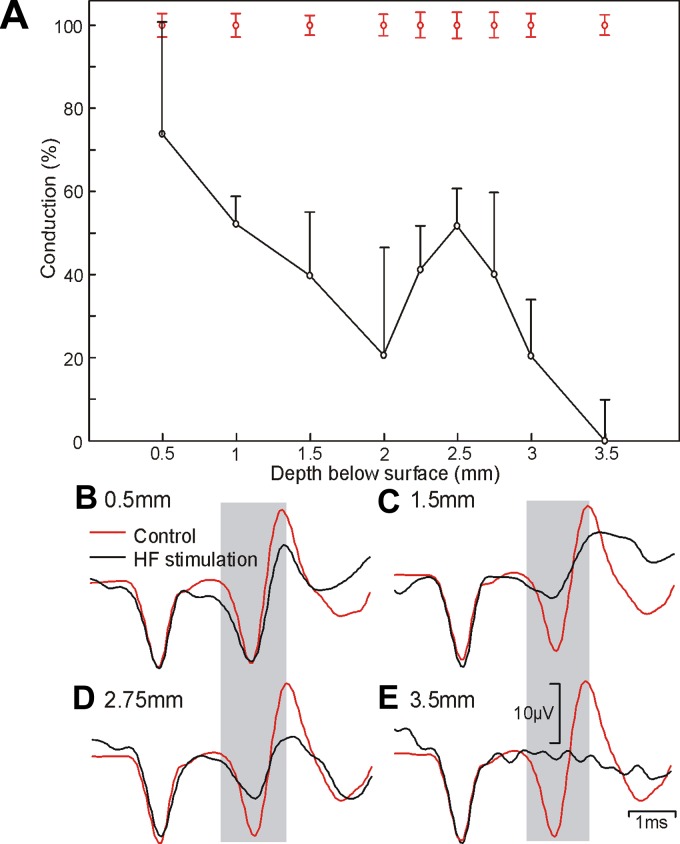
Spatial profile of HF conduction block. *A*: the amplitude of the antidromic corticospinal potential recorded from M1 elicited by the caudal spinal electrode during HF stimulation (1 mA) through the rostral electrode at different depths below the surface of the spinal cord. *B–E*: example traces recorded at the depths indicated. Red lines show responses to caudal electrode alone and black lines to stimulation during HF block. Data were recorded in *baboon L*.

**Fig. 4.**
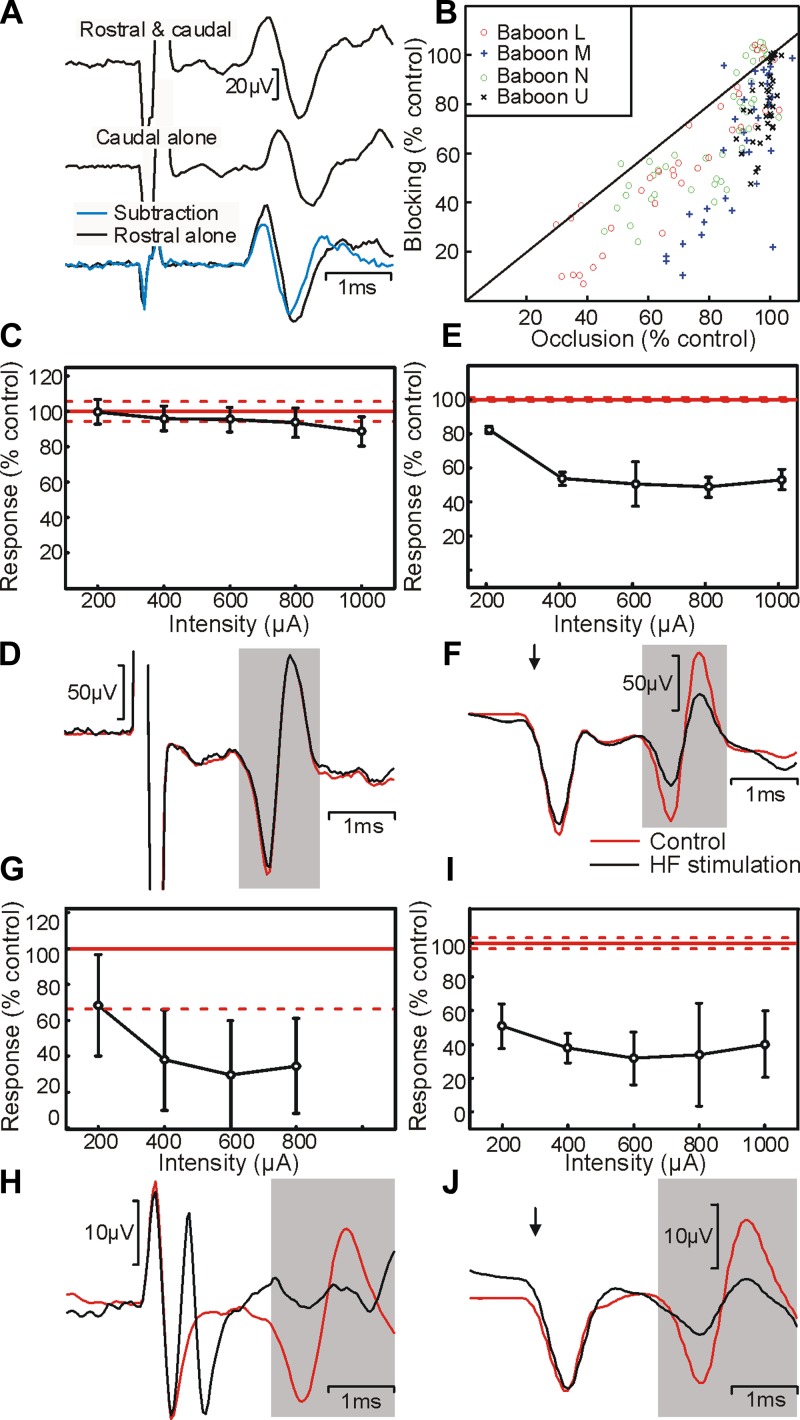
Relationship between block and stimulation. *A*: illustration of the method used to calculate overlap between fiber populations stimulated by rostral and caudal spinal electrodes. The response to stimulation of the caudal electrode alone was subtracted from the response to stimulation of both rostral and caudal electrodes simultaneously; this was compared with the response to the rostral electrode alone. *B*: scatter plot showing the amount of blocking vs. the amount of occlusion. Each point represents data from a different stimulus intensity and electrode location; different symbols show data from each of the 4 animals tested. Data points below the solid line indicate locations where the proportion of fibers blocked was more than that of those occluded. *C*: variation of occlusion with stimulus intensity to the rostral electrode. Points show the amplitude of the antidromic potential in the subtracted average as a percentage of the amplitude of the response to the rostral electrode alone (100%; solid red line). *D*: example traces related to *C*; black trace is with 1-mA sinusoidal stimulation and red trace is control. *E*: variation in amplitude of antidromic potential elicited from stimulation of the caudal electrode with amplitude of sinusoidal current at the rostral electrode. Amplitudes are expressed as a percentage of those seen without sinusoidal stimulation (100%; solid red line). *F*: example traces related to *E*; black trace is during 1-mA sinusoidal stimulation and red trace is control. Amplitude of the caudal electrode stimulus was 500 μA, and sinusoidal block was 2 kHz throughout. Results were recorded from *baboon U*. *G–J*: same as *C–F*, but for *baboon L*. Error bars and dotted red lines indicate means ± SE.

By applying these processing methods, it was possible to extract clear recordings of responses whose amplitude could be reliably measured. The effectiveness of the processing was validated by the numerous instances when for whatever reason sinusoidal block was ineffective, and we recovered responses after artifact correction very similar to control waveforms (see for example Figs. 2, 3*B*, and 4*D*). All data analysis was performed in the MATLAB programming environment.

## RESULTS

### 

#### Effect of frequency and intensity of sinusoidal current applied to the dorsolateral funiculus.

High-frequency sinusoidal stimulation was able to block conduction within the corticospinal tract of all animals tested. In each case, we found a frequency- and intensity-specific effect of blocking cortical field potentials recorded over M1. An example data set is shown in Fig. 2. The most effective stimulus frequency was 2 kHz, which was able to block the majority of the antidromic response at the highest intensity in three of the four animals (93.5, 99.6, 47.2, and 73.2% of response blocked at maximum intensity for *baboons M*, *L*, *U*, and *N*, respectively). There was a positive relationship between blocking efficacy and HF stimulus intensity; however, this grew weaker as frequency increased.

#### Spatial extent of block and comparison with stimulation.

The depth profile illustrated in Fig. 3 shows how blocking changed as the movable electrode was advanced into the spinal cord of *baboon L*, keeping the intensity and frequency of the sinusoidal stimulus the same (1 mA, 2 kHz). A clear increase in conduction block was apparent as the electrode was advanced into the cord, although this appeared to have two distinct phases. The first phase produced a peak in block at a depth of 2 mm; this is consistent with the tip lying in a central region of the dorsolateral funiculus. As the movable electrode was advanced deeper into the cord, there was a second, more pronounced period of block at 3.5 mm. We suggest that this could reflect stimulation of a medial area of the white matter that borders the intermediate zone. This region has recently been shown to be densely populated with corticospinal fibers that are coursing into the gray matter to synapse onto interneurons (Rosenzweig et al. 2009).

Figure 3 makes clear that HF blocking is only effective within a limited distance of the electrode tip. We were therefore interested in comparing the ability of a sinusoidal current to block a population of axons with the ability of a square current pulse through the same electrode to stimulate them. We estimated the latter using an occlusion test, illustrated in Fig. 4*A*. The response to stimulation of both rostral and caudal spinal electrodes simultaneously was subtracted from the response to the rostral electrode alone, yielding the additional fibers activated from the caudal electrode. This was compared with the response to stimulation of the caudal electrode alone. If there was no overlap between the two populations of stimulated fibers, these two traces would be the same. If there was complete overlap, the subtracted trace would show no response. Measuring the amplitude of the subtracted trace as a percentage of the amplitude of the response to caudal electrode alone thus quantified the extent of overlap, i.e., how many of the fibers activated by the rostral electrode could also be activated by the caudal electrode.

When comparing overlap of activation produced by stimulation with extent of block, we found two distinct patterns (each seen in 2/4 animals). For the experiment illustrated in Fig. 4, *C–F*, there was limited occlusion. The rostral electrode seemed capable of activating only a small fraction of the same corticospinal axons as the caudal electrode (Fig. 4, *C* and *D*, 11.3% at 1-mA intensity). However, a 2-kHz sinusoidal current passed through the rostral electrode could block around 50% of the response activated by the caudal electrode, even with a sinusoidal amplitude as low as 400 μA (Fig. 4, *E* and *F*). By contrast, for the experiment illustrated in Fig. 4, *G–J*, there was substantial occlusion. Stimulation through the rostral electrode was capable of activating 65.4% of the same response as the caudal electrode at the maximum intensity tested of 800 μA (Fig. 4, *G* and *H*). Sinusoidal currents passed through the rostral electrode blocked the majority of conduction at this amplitude (Fig. 4, *I* and *J*). In all animals, we found that HF stimulation typically blocked a greater proportion of fibers than could be activated by stimulation at the same intensity (Fig. 4*B*).

#### Onset time course.

**Fig. 5.**
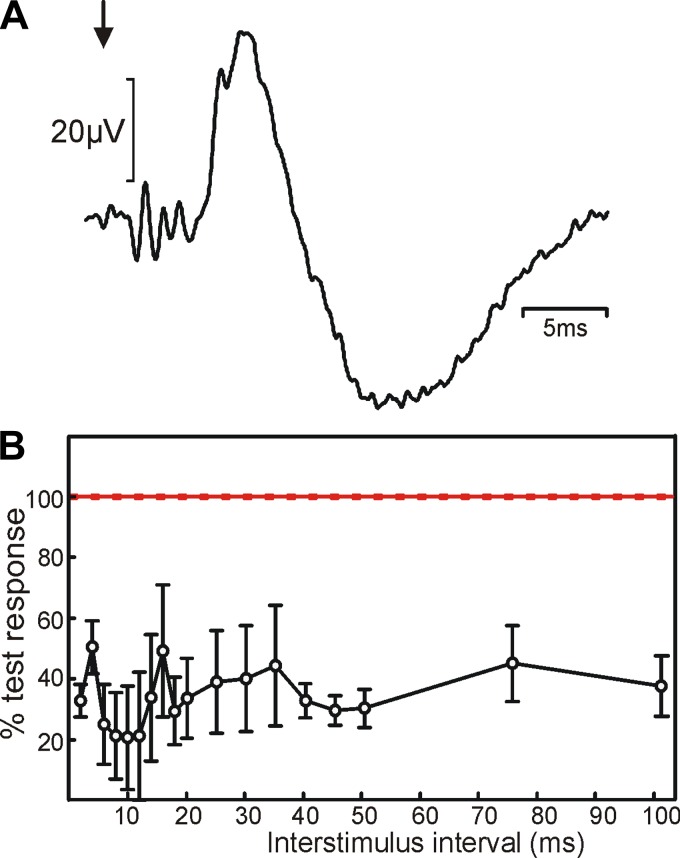
Block time course. *A*: average of the M1 epidural response following onset of the HF sinusoid (arrow). *B*: amplitude of the antidromic potential elicited from the caudal spinal electrode as a function of the time of the stimulus after the onset of sinusoidal stimulation to the rostral electrode. Amplitudes are expressed as a percentage of the response to stimulation of the caudal electrode alone (100%; solid red). A 1-mA stimulus was given through the caudal electrode and a 1-mA, 2-kHz sine wave through the rostral electrode throughout. Data were recorded in *baboon U*.

We examined the time course of the onset of conduction block in one animal; results are shown in Fig. 5. For this experiment, the moveable electrode was placed at the site of maximal block and a 2-kHz, 1-mA sinusoid was used throughout. Stimuli were delivered at different intervals after the onset of the sinusoidal current. For each interval to be tested, stimuli were tested on alternate trials at this interval and an interval 0.25 ms longer (half a cycle at 2 kHz) to ensure maximal cancellation of the sinusoidal artifact in averages (Fig. 1). As well as the stimulus artifact, the onset of the sinusoidal current elicited a large physiological response in the M1 recording (Fig. 5*A*). The response to sinusoidal current alone was subtracted from each sweep before the antidromic response produced from the caudal electrode was measured.

There appeared to be two distinct phases to the time course of blocking, as demonstrated previously in peripheral nerve. The first phase had an immediate onset and was reduced even when going from the 2- to 4-ms interval. This is likely to reflect activation of corticospinal fibers around the onset of the HF sinusoid. The elicited volley will show occlusion with that produced by the caudal stimulating electrode and then leave axons in a refractory state and unable to conduct. It is therefore perhaps slightly misleading to refer to this phase as “block,” since the fibers were activated by the sinusoidal current and simply could not be activated again. Intervals >6 ms produced a more sustained phase of reduced response that is likely to represent true conduction block of corticospinal fibers.

We did not examine the time course of recovery from block in detail. However, our protocol delivered the first “control” stimulus 500 ms after the offset of the HF sinusoid; the response to this stimulus was the same as that to later control stimuli, suggesting that recovery was already complete by this time, similar to previous findings in the peripheral nerve (Bhadra and Kilgore 2005).

#### HF sinusoidal stimulation of other central pathways.

**Fig. 6.**
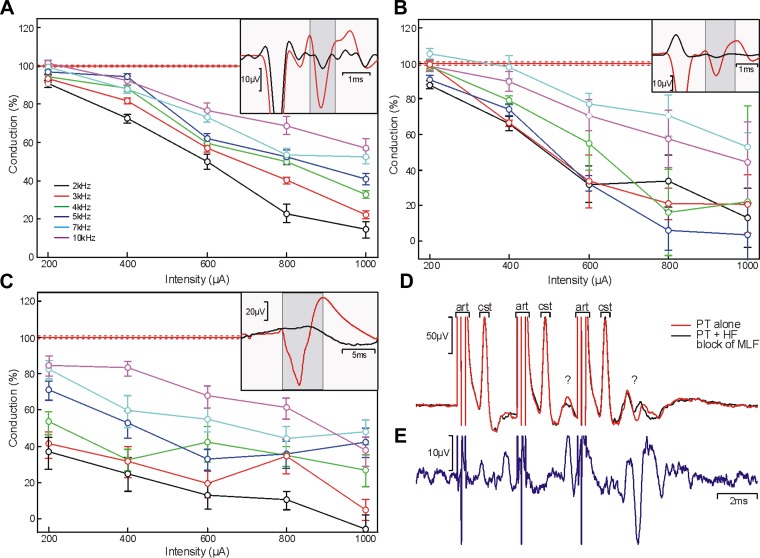
HF blockade of different pathways in the central nervous system. *A*: blocking protocol applied directly to the pyramidal tract (PT) through an implanted metal microelectrode. Stimulation was applied to the dorsolateral funiculus (DLF) at C5, and recordings were made via intracortical microwire electrodes. *Inset* plot shows averaged responses obtained with 2-kHz, 1-mA blocking stimuli; red trace shows stimulation of DLF alone and black trace shows stimulation of DLF during HF sinusoidal block of PT. *B*: repeat of PT block with square wave pulses at the same stimulus frequencies and intensities. *C*: blocking applied to the ascending dorsal column pathway within the spinal cord. Measurements were taken from somatosensory evoked potentials (SEPs) recorded in S1, elicited by stimulation of the contralateral median nerve (2-mA stimulus). *Inset* plot shows averaged responses obtained with 2-kHz, 1-mA blocking stimuli; red trace shows stimulation of median nerve alone and black trace shows stimulation of median nerve during HF block of dorsal columns. Data in *A–C* were recorded in macaque. *D* and *E*: use of HF blocking to reveal pathway contributing to a response of unknown origin. *D*: average cord dorsum response following stimulation of the PT at the medulla (1 mA, 3 shocks). art, Stimulus artifact; cst, corticospinal volley; ?, later transsynaptic response of unknown origin. Red trace shows response to stimulation of PT alone, and black trace shows stimulation of PT delivered during HF sinusoidal block of the medial longitudinal fasciculus (MLF; 1.5 mA, 2 kHz). *E*: difference between the red and black traces in *D*. The late response is reduced during HF block of the MLF, indicating that fibers passing through the MLF contribute to it. Data in *D* and *E* were recorded in *baboon P*.

We examined whether HF stimulation is also useful for blockade of other central neural pathways. First, we tested whether it was possible to block the corticospinal tract over its intracranial course by applying the blocking stimulus directly to the pyramidal tract at the medulla. As before, we generated an antidromic response in M1 by stimulating the cervical cord. We then applied HF sinusoidal stimulation (Fig. 6*A*) to the pyramidal tract. At 1-mA, 2-kHz stimulation, this was capable of blocking 85.7% of the antidromic response in M1.

Some laboratories may lack isolated constant-current devices capable of delivering sinusoidal stimuli. We therefore also tested whether block could be induced using square pulses. These were generated with the use of a standard experimental stimulator, set to deliver biphasic pulses with the width of each phase equal to half the cycle time. As shown in Fig. 6*B*, such square-pulse HF stimulation was also able to generate substantial block. However, whereas for sinusoidal stimuli the most effective frequency was the lowest tested (2 kHz), this was not the case for square-pulse HF stimulation. Pulses delivered at 5 kHz appeared to yield greater block than those at either 4 or 7 kHz (Fig. 6*B*).

We also tested whether HF stimulation was capable of blocking central sensory pathways. Stimuli delivered to the median nerve evoked a somatosensory evoked potential over sensorimotor cortex (Fig. 6*C*, *inset*, red). When HF sinusoidal stimuli were applied to the dorsal column of the spinal cord, a substantial reduction in the somatosensory evoked potential was seen (Fig. 6*C*). This was marked even at the lowest intensities, demonstrating a particular sensitivity of this pathway to disruption from HF stimulation.

As noted above, one frequent use of experimental lesions is to reveal which pathway underlies a neural response. To demonstrate that HF block is effective when used in this way, we recorded from the dorsal surface of the cervical spinal cord in one animal following stimulation through an electrode implanted in the PT at the medulla. As shown in Fig. 6*D* (red), when we delivered a train of three stimuli to the PT, each stimulus was followed by a large short-latency corticospinal volley on the cord dorsum. In addition, the second and third shocks elicited later responses, which grew from second to third shock. These responses are not likely to result from current spread to other central pathways, because we have previously verified that a 1-mA stimulus to the PT in a macaque only just spreads to the adjacent contralateral pyramid (Soteropoulos et al. 2011, Fig. 3*B*); spread is even less likely in the larger brain of a baboon. Rather, the late responses presumably reflect transsynaptic processes originating from stimulated corticospinal fibers, but there are many potential pathways. Possibilities include recurrent activation of corticospinal neurons in M1 by the antidromic volley (Jackson et al. 2002), activation by corticospinal collaterals of C3–C4 propriospinal interneurons (Isa et al. 2006) or reticulospinal neurons (Keizer and Kuypers 1989), and activation of segmental spinal circuits (Riddle and Baker 2010). We tested one of these possibilities by placing a second electrode within the MLF, which carries many reticulospinal axons, and applying HF block through this electrode.

Figure 6*D* shows results of this experiment, comparing results from trains of three stimuli to the PT electrode alone (red) and during HF block of the MLF (black). Whereas the early corticospinal volley was unchanged, there were clear reductions in components of the later potentials. This is made clearer in Fig. 6*E*, which presents the difference between the two traces of Fig. 6*D*. The results demonstrate the existence of a reticulospinal volley following stimulus trains to the PT in primate, in agreement with previous reports in cat (Edgley et al. 2004).

#### Long-duration block.

**Fig. 7.**
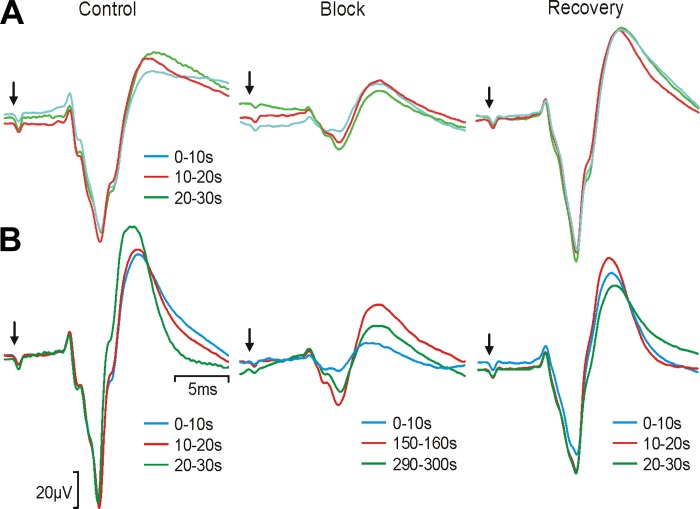
Application of long durations of HF block. *A*: 30 s of continuous HF sinusoidal block applied to the dorsal columns during recording of SEPs from the sensorimotor cortex. *Left*, average SEPs elicited by a 2-mA stimulus to the median nerve in successive epochs of 10 s (biphasic pulses, 0.2 ms per phase, 4 Hz, *n* = 40 stimuli). *Middle*, averages of responses corresponding to 0–10 s (cyan), 10–20 s (red), and 20–30 s (green) after block onset. *Right*, averages of responses corresponding to 0–10 s, 10–20 s, and 20–30 s after block offset. Arrows indicate time of median nerve stimulus. *B*: 5-min period of HF sinusoidal block applied to the dorsal columns. *Left*, initial average SEPs in response to median nerve stimulation, presented in successive 10-s epochs. *Middle*, average SEPs recorded at the start (cyan), middle (red), and end (green) of the 5-min period of HF block (numbers indicate the time in seconds following the start of HF stimulation). *Right*, average SEPs in 10-s blocks recorded after the HF sinusoidal stimulation was stopped. Each average corresponds to 40 stimuli, delivered at 4 Hz. Data were recorded in macaque.

The results presented above concerned brief HF stimuli lasting no more than 0.5 s. In a further experiment, we also tested whether block would continue during a longer stimulus and whether such stimulation would have effects that outlasted the application of HF current. Figure 7*A* shows somatosensory evoked potentials obtained before (*left*), during (*middle*), and after (*right*) 30 s of continuous HF sinusoidal stimulation to the dorsal column (1 mA, 2 kHz). Note that the somatosensory evoked potential had two phases: the initial, negative phase was very consistent between sweeps in the control period and likely reflects the earliest synaptic input to the cortex; the later, positive phase was more variable during the control period and probably reflects later cortical processing that depends on exact background state. Block of the first part of the response was maintained throughout the stimulation period, although there was a small decrease after 10 s had elapsed; this decrement did not seem to reflect any adverse effect, because the response returned to the control level rapidly after HF stimulation was terminated. However, during an even longer 5-min period of HF stimulation (Fig. 7*B*), we observed considerable variability that could reflect the onset of tissue damage. The first 10 s of prolonged HF stimulation produced a block equivalent to that shown in Fig. 7*A*, but this was reduced markedly thereafter. Furthermore, when HF stimulation ceased, the early part of the somatosensory evoked potential did not recover to baseline level, at least not over the 30-s recovery period that we monitored.

## DISCUSSION

We have shown that transient near-complete block of pathways in the central nervous system can be achieved with the use of a HF sinusoidal stimulus delivered through a single sharp metal microelectrode. This is a significant development because it demonstrates that principles carefully identified in the peripheral nervous system also apply to central pathways. As in previous work, we term the phenomenon a “conduction block.” Alternative explanations for the reduction in responses might involve synaptic depletion or antidromic collision. Synaptic depletion cannot explain the observed effects on antidromic potentials, and the fact that block could be sustained for 30 s but then recover rapidly suggests that the blocking electrode was not continually stimulating fibers. The technique has potential to be exploited for both experimental purposes and clinical benefit. Possible therapeutic applications include reducing symptoms such as pain and spasticity, which are otherwise notoriously difficult to treat and represent a high burden for both patients and society.

### 

#### HF block of the corticospinal tract.

Our results demonstrate a range of conduction block using HF sinusoidal stimulation. The maximum achieved was 99.6%, similar to the complete block reported in studies of peripheral nerves. However, in two of four animals we achieved <75% block of antidromic fast corticospinal conduction. Although under optimal conditions a complete block can be achieved, it is important to be aware that variation in electrode placement or inter-individual differences may lead to incomplete blocking in some cases.

Blocking depended on stimulus frequency; in all animals, we found that a 2-kHz sinusoid (the lowest frequency tested) was most effective. This contrasts with some previous work in peripheral nerve, which reported higher frequencies to be optimal (Tai et al. 2004); in particular, for primate peripheral nerve much higher frequencies (20–40 kHz) are required than for rodents and cats (Ackermann et al. 2011b), although the precise optimal frequency varies between individual animals (Bhadra et al. 2006). We were unable to test such high frequencies in the primate central nervous system because of the limited frequency response of our current delivery system. However, previous authors also have shown that block threshold increases with stimulus frequency between 1 and 30 kHz (Bhadra et al. 2006; Bhadra and Kilgore 2005; Joseph and Butera 2011; Kilgore and Bhadra 2004; Gaunt and Prochazka 2009), in agreement with our finding of reduced block as frequency increased. By using square rather than sinusoidal HF currents, we were also able to generate block, although the dependence on frequency appeared to show subtle differences, with 5-kHz stimuli being more effective than either 4 or 7 kHz; 2 kHz was also effective (Fig. 6*B*).

We did not test frequencies below 2 kHz because in preliminary recordings lower frequencies led to sustained activation, visible in animals not under neuromuscular blockade as repeated twitches. Even at 2 kHz there was a substantial transient activation of motor pathways (see Fig. 5*A*), leading to a large twitch if the animal was not maintained under neuromuscular block. Previous work in peripheral nerve has attempted to reduce the transient activation by using slowly increasing sinusoidal amplitudes, but without success (Miles et al. 2007). A paradigm that commences with a high-frequency (30 kHz) and high-current stimulus and then transitions to lower frequency with lower current (10 kHz) has been shown to minimize onset response in rat peripheral nerve (Gerges et al. 2010). Alternatively, HF stimulation can be combined with direct current (DC) to reduce the onset transient (Ackermann et al. 2011a; Franke et al. 2013), although in central pathways this would raise concerns about producing permanent lesions from the DC stimulus. It is possible that similar approaches could be used for onset transient suppression in central pathways, but there is likely to be a very sensitive dependence on the precise biophysics of the axons involved, and hence it would be necessary to tune the paradigm specifically for each application. In many cases, the existence of an onset response does not compromise the utility of the technique (e.g., Fig. 6*D*). Examination of the time course of blocking onset was made complex by the interaction with the onset transient response. However, it was clear that blocking was maximal as soon as this response started to decline (Fig. 5).

As well as testing central rather than peripheral axons, our study differed from previous work because HF current was delivered between a metal microelectrode insulated except for its tip and a distant reference, rather than a bipolar cuff electrode surrounding the nerve allowing focal current flow. Gaunt and Prochazka (2009) recently compared mono- and bipolar HF stimulation in cat pudendal nerve. Although bipolar stimulation required lower currents for block, blocking could still be achieved in the monopolar arrangement. Metal microelectrodes are typically used for experimental stimulation of the brain or spinal cord; the demonstration that HF block is possible using this configuration will allow straightforward integration of the technique into many studies.

Our main assay of conduction block was the antidromic field potential recorded over M1 following corticospinal stimulation in the cord. This will depend on only the fastest conducting fibers; we cannot comment on the impact of HF block on axons with slower conducting fibers, which are much more numerous (Humphrey and Corrie 1978). The somatosensory evoked potential that we also assessed is likewise dependent on fast-conducting fibers in the dorsal columns. In peripheral nerve, Liu et al. (2013) demonstrated that conduction in the largest fibers was blocked at lower threshold, and recovered later, than in smaller fibers. It is likely that a similar bias toward blocking fast fibers will occur in central pathways at frequencies ≤10 kHz. In addition, slowly conducting C-fibers in peripheral nerve show a nonmonotonic variation of block threshold with frequency, with a second blocking region at frequencies >40 kHz (Joseph and Butera 2011). Delivering currents at these higher frequencies may thus provide a means of selectively blocking only slow fibers, which could be valuable in some studies.

#### Future use of HF block.

High-frequency conduction block has the potential to become a useful experimental method. Unlike surgical lesions, it is quickly reversible. Protocols can therefore be repeated numerous times within one animal, leading to a reduction in animal numbers required for a given study. Although we see no reason why the method should not work in any central axon tract, it would be important for future studies to confirm the optimal frequency and intensity in the targeted structure, rather than assuming that the parameters that we have found to work in primate corticospinal tract and ascending sensory pathways are universally applicable. Although sinusoidal waveforms have been best investigated in peripheral nerve, rectangular waveforms (Bhadra and Kilgore 2004) and square wave pulses may also be effective (Fig. 6*B*), possibly opening the method to laboratories lacking equipment for arbitrary isolated current waveform delivery.

It is important to consider the safety of this technique as it is developed for further applications. Damaging effects of long-term electrical stimulation on nerve fibers have been reported (McCreery et al. 1995), and Liu et al. (2013) recently cautioned that HF stimulation can have long-lasting effects on nerve conduction, with the potential for nerve damage if used inappropriately. However, stimulus durations used in that particular study were substantially longer (5–10 s) than the 0.5 s used in most experiments here. Often for experimental use as illustrated in Fig. 6, *D and E*, only the briefest period of blocking is required, thereby lessening the chances of long-term damage. During our extended periods of HF stimulation of the dorsal column, we found complete recovery of somatosensory evoked potentials from a 30-s application but not following 5 min of block, underlining the potential for damage to central pathways following excessive stimulation. Gaunt and Prochazka (2009) showed stable blocking thresholds measured over 230 days when recording from cats implanted with cuffs on the pudendal nerve, suggesting that so long as blocking is kept within limits it has no cumulative long-term effects.

High-frequency conduction block is useful in vitro or for anesthetized whole animal preparations, where movements associated with the powerful onset transient activity can be blocked pharmacologically and there is no conscious perception of potentially unpleasant sensory activation. It would also be desirable to use the technique in the conscious state, either to make experimental lesions in behaving animals or as a therapy in patients, but onset activation is likely to present a severe limitation. Such activity has been reported only once in the literature in an awake cat (Gaunt and Prochazka 2009); the authors described a “mild aversive response” to HF stimulation of the pudendal nerve. Although this may be tolerable, the consequences of onset responses associated with stimulating central pathways are likely to be much more unpleasant, precluding use in awake subjects unless approaches to minimize onset response can be shown to work effectively in central axons (Ackermann et al. 2010a, 2011a; Franke et al. 2013; Gerges et al. 2010). For long-term chronic use, Gaunt and Prochazka (2009) described a system capable of delivering the sinusoidal current to implanted electrodes without the need for transcutaneous connectors or indwelling electronics. With minimal implanted material leading to reduced chance of postsurgical infection and a low risk of technical failure consequent on the simple design, this could have a number of uses as a clinical intervention if the problem of unwanted onset activity can be solved.

## GRANTS

This work was funded by Newcastle University. S. N. Baker is supported by a Wellcome Trust Senior Fellowship.

## DISCLOSURES

No conflicts of interest, financial or otherwise, are declared by the authors.

## AUTHOR CONTRIBUTIONS

K.M.F. and S.N.B. conception and design of research; K.M.F., N.E.J., G.O.O., and S.N.B. performed experiments; K.M.F. and S.N.B. analyzed data; K.M.F. and S.N.B. interpreted results of experiments; K.M.F. and S.N.B. prepared figures; K.M.F. and S.N.B. drafted manuscript; K.M.F., N.E.J., G.O.O., and S.N.B. edited and revised manuscript; K.M.F., N.E.J., G.O.O., and S.N.B. approved final version of manuscript.
